# An IoT Enable Anomaly Detection System for Smart City Surveillance

**DOI:** 10.3390/s23042358

**Published:** 2023-02-20

**Authors:** Muhammad Islam, Abdulsalam S. Dukyil, Saleh Alyahya, Shabana Habib

**Affiliations:** 1 Department of Electrical Engineering, College of Engineering and Information Technology, Onaizah Colleges, Onaizah 2053, Saudi Arabia; 2 STC Academy, Riyadh 13315, Saudi Arabia; 3 Department of Information Technology, College of Computer, Qassim University, Buraydah 51452, Saudi Arabia

**Keywords:** anomaly detection, ESN, CCTV, smart city, IoT

## Abstract

Since the advent of visual sensors, smart cities have generated massive surveillance video data, which can be intelligently inspected to detect anomalies. Computer vision-based automated anomaly detection techniques replace human intervention to secure video surveillance applications in place from traditional video surveillance systems that rely on human involvement for anomaly detection, which is tedious and inaccurate. Due to the diverse nature of anomalous events and their complexity, it is however, very challenging to detect them automatically in a real-world scenario. By using Artificial Intelligence of Things (AIoT), this research work presents an efficient and robust framework for detecting anomalies in surveillance large video data. A hybrid model integrating 2D-CNN and ESN are proposed in this research study for smart surveillance, which is an important application of AIoT. The CNN is used as feature extractor from input videos which are then inputted to autoencoder for feature refinement followed by ESN for sequence learning and anomalous events detection. The proposed model is lightweight and implemented over edge devices to ensure their capability and applicability over AIoT environments in a smart city. The proposed model significantly enhanced performance using challenging surveillance datasets compared to other methods.

## 1. Introduction

**Figure 1:**
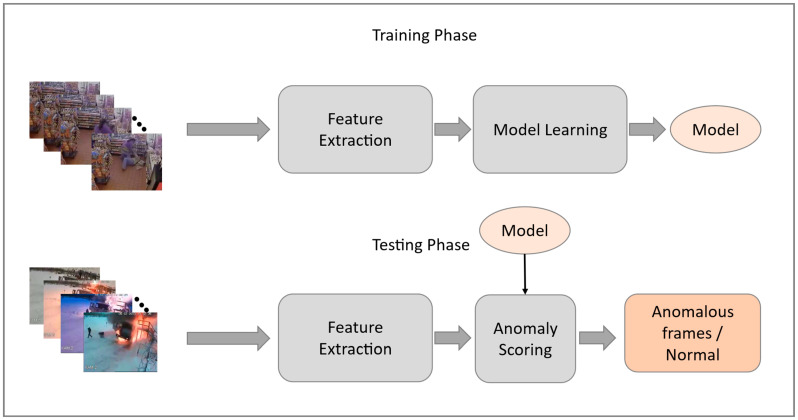
General pipeline of anomaly detection.

There have been more human lives and property losses as a result of crimes in the 21st century than any other human-centered issue [1]. A surveillance system that is capable to automatically detect and report abnormal behavior is one of the most notable solutions for early detection of unusual behaviors indoors and outdoors. Public areas, such as shopping malls, airports, parks, and so on, are equipped with an increasing number of distributed surveillance cameras to ensure public safety and security [2]. By using computer vision techniques, we can detect unusual events automatically and efficiently, however, manually analyzing huge surveillance video data are laborious, inaccurate, and time-consuming. There are some challenges involved, such as illumination variation, people’s appearance, and perspective distance from a camera. Consequently, intelligent surveillance techniques which are capable of detecting unusual events at an early stage and alerting the appropriate departments, are in a great demand in the current technological era. A general pipeline of anomaly detection in video is given in Figure 1.

A surveillance video can detect anomalous events when targets overlap, frames are crowded, targets are partially or completely occluded, noise is present, and things are handled badly. From the input visual data, anomaly detection becomes more complicated when vision sensors cover a significant number of entities, including anomalous targets and normal people.

Furthermore, anomalous events are unpredictable, occur infrequently, and have an unclear definition, so collecting all unusual samples and clearly defining anomaly for an AI model is challenging due to their unbounded nature, infrequent occurrence, and unclear definition. For instance, a running person can be considered normal on one field (soccer field) but ambiguous on another (shopping mall). Surveillance video data can be compiled more easily when it contains usual events rather than anomalous ones. The act of anomaly detection involves detecting occurrences and events, objects, and behaviors that are very unlikely in comparison to the normal events in the world. Furthermore, there are various types of abnormalities that can be detected in abnormality detection, which can be categorized as universal anomalies [3,4]. There are a bunch of traditional feature-based anomaly detection techniques [5], but they’re not effective for complex surveillance scenes because they’re limited in capability [6]. There is no doubt that existing surveillance abnormality classification systems are flexible, but they are highly domain-specific, which means that they are not capable of classifying all sorts of surveillance anomalies [7].

AI models are often utilized in anomaly detection domain to identify anomalies in videos, which has been a subject of many researchers. AI techniques are classified into three categories based on the variables of the training sets: unsupervised-, supervised-, and semi-supervised-systems [8]. It has been shown that frame reconstruction, future prediction-based, and clustering, methods are effective in finding anomalies when labels are not provided in the training datasets [9,10]. As far as dealing with surveillance video data from the real world is concerned, these techniques reveal limited performance. In supervised learning abnormal and normal data are included in the training set to address these limitations. In particular, weakly supervised techniques can help solving anomaly detection problems in a more compared manner than their strongly supervised counterparts because they include only video-level labels as part of the training set for both normal and anomalous events respectively [11]. The clips that make up a video in MIL are viewed as instances, while the video itself is considered a bag where annotations at the bag level are used to learn anomaly labels at the instance level. Anomalies are defined as occurrences that differ from predicted normal behavior, which allows semi-supervised methods to be advantageous [12]. The term anomaly is used to describe data which deviates from the usual pattern or data set of results as an outcome of a particular event.

There have been several investigations in the past that have addressed semi-supervised anomaly detection. There are several techniques available to address this problem, but the basic idea is to generate a model or representation that captures both visual appearance and normal motion patterns [13]. Several researchers, such as [14,15], have employed the motion trajectories of the objects of interest to deliver information about the normal or expected patterns of the objects being examined [16].

Any deviations from the expected patterns are termed outliers, which refers to anomalies in terms of the work method. Because trajectory-based techniques focus only on visual patterns and ignores the importance of targets in complex situations such as crowded scenes, they show limited performance. Furthermore, Dictionary learning or sparse coding are also prominent video anomaly detection techniques [17,18]. This type of approach encodes regular events into a dictionary, and input patterns that are not found in the dictionary which are considered to be abnormal. A trained model generates normal and anomalous events depending on the size of the reconstruction error during the testing phase [16]. Due to the variability of normal patterns, different weather conditions, lighting situations, etc., these approaches suffer from a high false alarm rate and an enormous amount of time spent optimizing sparse coefficients [19].

To overcome the above-mentioned problems, this research paper is an instant artificial intelligence-assisted anomaly detection system is introduced which can be suitable for resource-constrained Edge devices. In order to address the computational complexity issue of existing methods. The lightweight nature of the proposed model is applicable to perform all processing over edge devices without considering additional servers. First frame-level features are extracted via an EfficientNet backbone which is efficient and effective compared to existing models. These features are then forwarded to the autoencoder for feature refinement. The refined are then inputted into ESN architecture for anomalous event detection. To assess the potential of our proposed network, authors perform extensive experiments using the UCF, ShanghaiTech, and surveillance fight datasets. As compared to existing modeling alternatives, results demonstrate competitive predictive performance and lower computational complexity.

The rest of the paper is organized as follows: Section 2 overview recent baseline approaches for anomaly detection, Section 3 describes the proposed methods, Section 4, report the experimental results, and finally Section 5 conclusion.

## 2. Related Work

For precisely identifying abnormal events in surveillance videos, several approaches have been proposed so far. Many techniques for detecting anomalies have been developed in the literature [20]. The unusual nature of events is often classified as “events that are deviating from normal patterns” in the majority of these works. Taking this description into consideration and when providing a prior information regarding the anomalies, it is often considered that the task of detecting abnormal events is essentially a classification task [21,22] in which visual features are analyzed and compared to classifier models for determining which activities are normal and which are abnormal.

### 2.1. Hand Crafted

There was a great deal of interest in the use of handcrafted features, including trajectory, sparse, and dense features, in early surveillance environments for detecting anomalies and violence, aggression, and accidents [23]. In tracking techniques, trajectory features represent the information for the path taken by moving objects. To categorize normal and abnormal movement trajectories, these models are trained first on normal movement trajectories. Using trajectories to identify anomalous patterns in low- and medium-density crowd video, in other study [24] authors present a real-time technique for detecting anomalies in low- and medium-density crowd video. Furthermore, in [4], relationships between Spatial-Temporal Interaction Points (STIPs) were exploited to determine what factors cause global anomalies, such as abnormal interactions among humans. Furthermore, a graph-based model of STIP interaction was developed by Singh et al. [25]. By using sparse reconstruction costs, Cheng et al. [26] utilize low-level features across STIPs to detect local and global anomalies. In a study [27], it was suggested that swarm intelligence in conjunction with HOG descriptors, would be used in conjunction with motion, appearance, and STIP detection techniques to capture the motion and appearance of nearby humans such that STIP detection could be performed. This approach has a significant limitation, and that is when the number of identified STIPs is small or large, meaning that when the number of movements in the video are too small or too large, the representativeness of events is not sufficient to represent the events accurately.

Comparing sparse and trajectory predictors to dense feature descriptors, complex and crowded scenes are often deemed more suitable for dense descriptors. Using the histogram of optical flow and gradients features, Zhang et al. [28] proposed a hybrid feature-based model. A follow up approach is presented in [29] where the authors developed a 3D method for extracting the entropy, motion, and appearance information from a specific scene by using a Histogram of Optical Flow Orientation and Magnitude and Entropy. In addition to experiencing severe limitations, dense feature extraction methods also utilize a lot of memory and require a lot of computation time. Furthermore, a complex and crowded scene limits the effectiveness of these techniques. Additionally, different scenes show differing numbers of STIPs, therefore they cannot produce accurate representations. Furthermore, detecting anomalies using these methods requires a great deal of memory capacity and computation time. Therefore, anomaly detection in video streams has been dominated by Deep Learning approaches over the last few years due to these downsides.

### 2.2. Deep Learning

A variety of deep neural networks have been developed over the last decade by several researchers in order to achieve impressive results [30]. The detection of anomalies in surveillance environments can be achieved by using deep neural networks and multi-instance learning [22]. A discriminative anomalous clip miner was proposed by Sun et al. [31], which examines segments that are discernible from normal segments which are anomalous. There is another model that combines multiple instance learning (MIL) ranking with temporal context in order to learn motion features with the use of an attention block to take into account the temporal context [32]. A 3D Siamese one-shot model for anomaly detection has been introduced by the researcher [33]. For the detection of video anomalies in a surveillance system, another study utilized CNN features combined with a multilayer bi-LSTM model without any further classification mechanisms [4]. Furthermore, several deep learning based methods are developed for the anomaly detection in recent literature including memory augmented network [34], Deep CNN [35], encoder-decoder model [36], deep temporal autoencoder [37], graph neural network [38], etc.

However, the performance of these methods are still questionable and required powerful servers to run these models. Therefore, this research work we developed an efficient and effective framework for anomaly detection.

## 3. The Proposed Method

**Figure 2:**
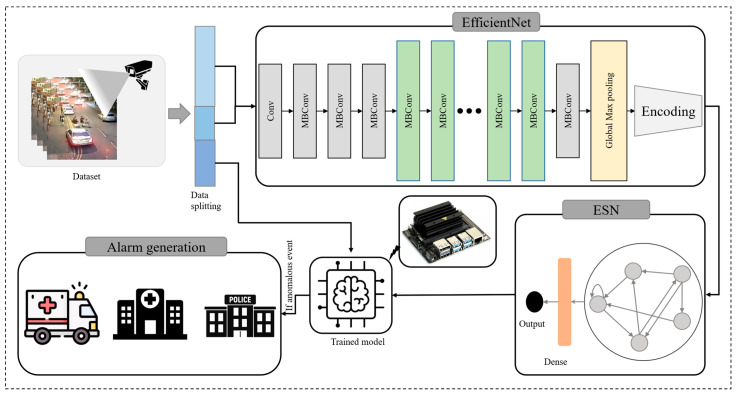
The proposed framework for anomaly detection.

Anomaly detection in surveillance refers to the process of identifying unusual or abnormal behavior in a surveillance system, such as security cameras or sensor networks. This can include identifying objects or individuals that are out of place, detecting unusual patterns of movement, or identifying unusual activity in a specific area. Anomaly detection can be used to improve security and reduce the risk of crime or other unwanted activities. Several methods are developed for anomaly detection include machine learning algorithms, however the performance of these methods further need to be improve for an effective anomaly detection system. Therefore, we developed a hybrid model combining CNN, autoencoder and ESN. The CNN was integrated to extract frame level feature which are then passed to autoencoder for feature refinement and finally fed to ESN for anomaly detection. A high-level diagram of the proposed model is given in Figure 2, where the proposed framework is also developed to generate alarm if any anomalous event detected and will ultimately notify the corresponding department for rescue and prevention according to the type of anomaly. The proposed model is lightweight and implemented over edge devices to provide a safety and secure smart surveillance to the users. The following section discusses each step of the proposed framework in detail.

### 3.1. Feature Extraction

The literature describes a variety of CNN-based models for fire detection [39,40], medical images [41], classifying videos [8,42], predicting time series data [43,44,45], forecasting [46], etc. Several CNN architectures have been used for feature extraction in recent literature, including EfficientNet [47], Squeeze Net, Google Net, and MobileNet, among others. This aims to boost their accuracy by modifying width, depth, or resolution of CNN-based architectures using different scaling strategies. In this regard, we investigated the EfficientNet [39] feature extraction, which utilizes compound scaling to scale all dimensions of the network. In order to optimize FLOPs and accuracy, EfficientNet uses multi-objective architecture search [48]. A search space of Tan et al. [49] has been utilized in this architecture. The hyperparameters such as network width and target FLOPs control the trade-off between accuracy and FLOPs in model, whereas this research work represents the FLOPs target, and T represents the accuracy target. In the EfficientNet architecture, there are several convolutional layers, each with a different number of kernels [50]. There are three inputs in this architecture, R, G, and B, and the size of the inputs is 150 × 150. In order to reduce feature map size, the hidden layers are scaled down to reduce the map size, but the network width is scaled up to improve accuracy, ensuring that the most important features are extracted from the input data. Upon receiving these features, they are then sent to autoencoders, which encode them in order to select the best optimal features.

### 3.2. Autoencoder

**Figure 3:**
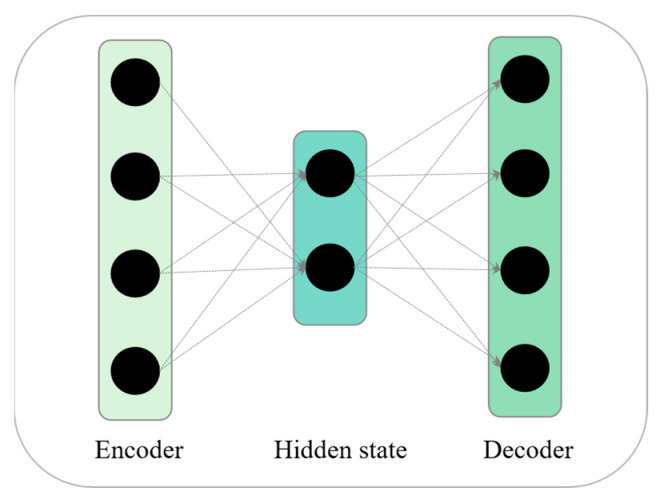
Internal architecture of autoencoder.

Unsupervised input in a feature map is typically understood using autoencoder-based architectures. There are three main layers in an autoencoder, namely encoder, hidden state, and decoder layers, which are shown in Figure 3. The main components of autoencoders are encoder and the decoder, where the encoder is used to reduce the input dimension while the decoder is used to renovate it. In the encoder, the input dimension is reduced, and, in the decoder, it is renovated. However, a mathematical equation is proposed in two parts, one which is the input data as far as authors interest is concerned, the other which is the output data decoder. As a matter of fact, the mathematical formulation for the output decoder can be found in the following Equations (1) and (2) [39].

(1) $$h_{n}=f\left(w_{1}x_{n}+b_{1}\right)$$

(2) $$o_{n}=G(w_{2}x_{n}+b_{2})$$

A network analysis can be performed when f represents an encoder function, G represents a decoder function, $w_{1}$ and $w_{2}$ represents a weight metric, and $b_{1}$ and $b_{2}$ are the bias term in case of a multi-encoding function. A compressed feature representation of the input data is created in the encoder part of the autoencoder by encoding the input data into the compressed format. As a result, the compressed features of an autoencoder are decoded and reconstructed with the aid of a decoding component. Encoding involves transforming the high-dimensional input features into a lower-dimensional representation that contains all of the input features in a different order.

### 3.3. Echo State Network

Deep neural networks with multiple layers architectures have received considerable attention by researchers in the field of neural networks in the last few years, as they contain more than one layer of data Goodfellow et al. [51]. Besides the hierarchical standardized RNN, the hierarchical RNN has also played an essential role in a number of complex problems, such as supervised learning and deep learning. Accordingly, in [52] the ESN was first combined with deep learning frameworks, which is computationally intelligent compared to other RNN variants since the ESN is a new and special type of RNN. According to Jaeger et al. [53], RNN is modeled by a reservoir that provides an essential architecture as well as supervised learning capabilities [54]. This ESN architecture consists primarily of three components: a reservoir, an input and an output [54,55]. The input unit is referred to as I, indicating the input layer, the reservoir is referred to R and the output unit is referred to O notations. The mathematical formulation if an ESN is given in Equations (3) to (8) [55].

(3) $$u\left(i\right)={\left[u_{1}\left(i\right),\;u_{2}\left(i\right)\ldots .u_{I}\left(i\right)\right]}^{t}$$

(4) $$x\left(i\right)={\left[x_{1}\left(i\right),\;x_{2}\left(i\right)\ldots .x_{R}\left(i\right)\right]}^{t}$$

(5) $$y\left(i\right)={\left[y_{1}\left(i\right),\;y_{2}\left(i\right)\ldots .y_{O}\left(i\right)\right]}^{t}$$

(6) $$y\;\left(i+1\right)=f\left(W_{in}\times u\left(i+1\right)+W\times x\left(i\right)+W_{back}\times y\left(i\right)\right)$$

(7) $$y\;\left(i+1\right)=g\left(W_{out}\times u\left(i+1\right)\right)$$

(8) $$W_{out}={\left(M^{-1}\times T\right)}^{t}$$

For both output and reservoir units in Equations (6) and (7), ${\;}^{\prime }f^{\prime }$ and ${\;}^{\prime }g^{\prime }$ indicate activation functions. During the learning process, the weight matrix remains unchanged while the reservoir matrix updates [54,55]. The readout weights for each target output are calculated based on the reservoir state vectors and target outputs. A time step equal to or greater than the output is used by Equation (8) in order to calculate the readout. These reservoirs are mainly responsible for the overall performance of the ESN, and they have the potential to influence the overall performance of this network through their influence on its three main parameters [55]. Moreover, a second investigation has found that the number of reservoir neurons plays an important role in the performance of ESN. To explain this, it is important to keep in mind that the internal structure of ESN is closely linked to the information regarding its hidden state. Also, other factors have to be taken into account, such as the quantity of training data that must be collected and the complexity of the targeted tasks. In contrast, the performance of the ESN is also influenced by the rate of connectivity, by the absolute eigenvalue of the weight matrix, and by the special radius, which is indicated at intervals between zero and one. Concluding that ESNs matter more than RNNs when it comes to the learning and approximation functions.

### 3.4. Architecture

In order to obtain frame-level information from input dataset, EfficientNet is used followed by an autoencoder for optimal feature selection where a resize of 150 × 150 × 3 is applied to each frame of the video. The extracted features are then reshaped with 30 frames to form a single second sequences. These sequences are then passed to ESN for feature learning and anomaly detection. The EfficientNet is also used to extract spatial features from each frame in the video. However, there is a very small change in each consecutive frames making the these features redundant. Therefore, autoencoder is used for optimal feature selection. The ESN is then employed to learn temporal dependencies in the data. Once the model is trained, it is then tested over test data. The proposed model is lightweight and applicable over edge devices as given in the result section. The lightweight nature of the proposed model making it able to be implemented over AIoT assisted surveillance system for smart cities. A common wireless sensor network is used to link vision sensors and IoT devices to broadcast alerts to other devices in the IoT network and generate alarm as soon as an event occurs.

## 4. Results

Using real-world surveillance datasets, we compared the proposed framework’s performance with the current state-of-the-art. AUC (Area Under Curve) and accuracy are used as evaluation criteria for the system. By utilizing surveillance datasets including UCF-Crime, ShanghaiTech, surveillance fight and non-surveillance datasets including Violent flow and hockey fight the proposed framework, outperform the existing anomaly detection techniques. The implementation is performed in Keras with backend TensorFlow using RTC 3070 GPU. The purpose of testing the proposed anomaly detection framework, we used two metrics, which are typically used for fair comparison by the state-of-the-art (SOTA) [22,32,56]. AUC is one type of statistical evaluation protocol that is used. The other type of statistical evaluation protocol is accuracy.

### 4.1. Datasets

We evaluated the proposed framework using UCF [22], ShanghaiTech [39], and Surveillance fight [57] datasets. It is important to note that these datasets are large-scale and challenging in terms of detecting anomalies and violence. The UCF-Crime database contains 14 different anomalies classes and 1900 videos of real-world situations that were all taken in UCF to evaluate the crimes committed [22]. Based on the baseline research, there are 810 normal videos and 800 anomalous videos in the training set, while 150, 140 normal and anomalous videos are in the training and testing set, respectively. The framework in this study was also evaluated on the ShanghaiTech [39] dataset, which has 437 videos with 130 anomalous events across 13 scenes. An indication of anomalous occurrences was provided at the frame level, along with ground truth at the pixel level. According to the procedure [56], the training set consists of 175 normal films and 63 anomalous films; the testing set consists of 155 normal films and 44 anomalous videos. From 15 to nearly one minute long, the video clips range in length. Akti et al. [57] have introduced a surveillance fight dataset that includes all types of fight videos, including both violent and non-violent ones. As a part of this dataset, we have comprised equal numbers of videos in each study group and in total there are 300 videos with a variety of resolutions.

As part of the evaluation, the proposed framework is compared with non-surveillance benchmark data for violence detection, such as the Hockey Fight dataset [58] and the Violent Flow dataset [59]. Hockey Fights is a dataset that covers violence events occurring during hockey matches within the National Hockey League. This dataset contains 1000 supervised video clips, 500 clips in each category, and it is labeled as fight or no fight, with a well-balanced number of clips in each segment. The violence dataset comprises five different sets of videos, which cover a wide range of violence and non-violence which include a total of 246 clips where each class include 246 videos. The video clips range from 25 to 200 frames in length, with a 320 × 240 resolution. Mobile phones or static cameras are used to capture video clips in these non-surveillance datasets.

For training and testing purposes the datasets are split into the following training, validation, and testing sets, however, the UCF and ShanghaiTech have training and testing set, therefore we selected 20% of data from training set for model validation. Furthermore, the Akti, Hockey fight, and violent flow datasets are split into 50% training, 20% validation, 30% testing for fair evaluation of the model with state-of-art models [14]. Furthermore, the proposed model and other ablation study models are trained on the training data and evaluated on testing data individually.

### 4.2. Comparative Analysis with Baselines

We compared the performance of the proposed framework with that of various SOTA approaches as given in Table 1, Table 2 and Table 3. The proposed architecture outperformed the recently spotted techniques by applying challenging anomaly datasets. We have used ShanghaiTech dataset and compared the propsoed model performance with various methods such as predictions of normal frames based on anomaly detection techniques with unsupervised learning [9,17], feature patterns based on unsupervised learning [60,61], and skeleton patterns based on unsupervised learning [62,63]. As a result, unsupervised techniques achieved lower siperfromance which was compared with supervised ones, since abnormal videos aren’t given in the training data, thus, the performance of these methods are lower than supervised techniques. In comparison, we have achieved the best results with the propspoed framework proposed compared to [56,62,64]. In addition, the UCF-Crime dataset was used to compare five state-of-the-art methods. AUC of 87.55% is achieved by the proposed framework, which was significantly higher than the AUCs of [21,22,56,65,66] indicating an increase of 1.73% over the previous research studies. A comparison of the experimental results with existing approaches showed an outstanding performance. In addition to this surveillance dataset, a more recent SOTA benchmark Surveillance Fight was also used to test the proposed model, and the experimental results were compared with recent approaches like SOTA [57,67,68]. With 95.8% accuracy, the proposed framework achieved best results when compared with the existing frameworks [57,67,68], which were improved by 2.7%, when compared with the recent studies [8].

As shown in Table 4 and Table 5, the proposed model offers significantly better performance than SOTA, even using non-surveillance datasets. Both indoor and outdoor surveillance results confirmed the effectiveness of the proposed framework. In comparison to recent techniques, this reserch work has achieved an increase of 0.8% accuracy with the proposed framework against [30,40,48,51,52,53,54,55,56,57]. Compared to the existing frameworks on violent flow, the proposed framework shows a 0.8% increase in accuracy compared to [8,57,67,68]. Experiments on large scale anomaly detection datasets with the proposed models revealed an outperformed existing techniques by a wide margin.

To Conclude the above results, The proposed network shows an improvement over existing state-of-the-art models for anomaly detection in video data due to several factors. Initially, the use of EfficientNet as a feature extractor ensures that the spatial information from each frame is effectively captured, while the use of an autoencoder further refines the extracted features by reducing the redundancy and retaining only the most important information. Additionally, the use of an Echo State Network (ESN) allows the model to learn the temporal dependencies in the data, providing a more comprehensive understanding of the input video sequence. The lightweight nature of the proposed model, which allows for its implementation over edge devices, is also an important factor contributing to its improved performance. By performing all processing over edge devices, the network avoids the computational overhead and latency associated with sending data to a central server for processing. In summary, the combination of efficient feature extraction, optimal feature selection, and temporal learning makes the proposed network an effective and efficient solution for anomaly detection in video data, with improved performance compared to existing state-of-the-art models.

### 4.3. Ablation Study

**Figure 4:**
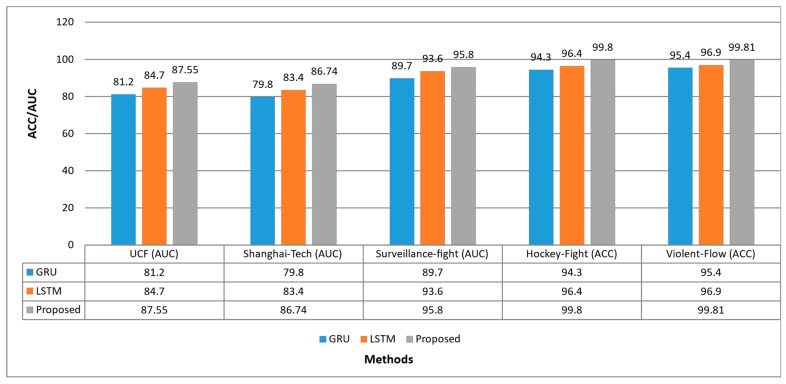
Performance comparison of the proposed model with ablation study methods.

The proposed model achieved considerable performance compared to state-of-the-art as given in Table 1, Table 2, Table 3, Table 4 and Table 5. However, prior finalizing the proposed architecture a supportive ablation study is performed. The ablation study is based on evaluating the performance of GRU, LSTM and the proposed ESN. The detailed results of these methods with backbone feature extraction and autoencoder are given in Figure 4. The Table concluded that GRU achieved lower performance compared to LSTM and ESN while LSTM achieved the second performance over all datasets.

### 4.4. Time Complexity

It is difficult to run computationally expensive models on the current surveillance systems due to their limited computational capabilities. To extract meaningful patterns from these videos, the domain experts and researchers transmit them to local or cloud servers. This often results in a slower response time and countermeasures because visual data is transmitted over a large bandwidth. This research work aims to develop a lightweight model for efficient anomaly detection to utilise the limited processor capabilities and memory of current surveillance sensors. We evaluate and compare the performance of the proposed model in two different settings such as GPU and Edge device (NanoJetson). According to the proposed model, 3.5 and 1 sequence are processed, respectively, over these settings. As a result, the proposed model process one sequence which has 30 frames over edge devices. Processing of 30 frames sequence in one second could be enough for real-time implementation over an edge device.

## 5. Conclusions

This research work, we developed an efficient and effective framework for anomalous event detection, which can be used both in non-surveillance and surveillance environments. The proposed framework includes feature extraction, feature refinement, and feature learning modules. For feature extraction we used the EfficientNet model can extract more robust information compared to other CNN variants. The extracted features are then forwarded to the autoencoder to select the best optimal features. These features are then forwarded to ESN for sequence learning and anomaly detection. Ultimatly, the proposed model an ablation study was also conducted. The results indicate that the proposed model achieved higher performance compared to state-of-the-art methods. The proposed model improve 1.73%, 1.84%, and 2.1% AUC over UCF, ShanghaiTech, and surveillance fight datasets, respectively. Furthermore, the propsoed model improved 0.5% and 0.8% accuracy over Hockey Fight and Violent Flow datasets. Alongside higher accuracy, the proposed model is also computationally inexpensive and is implemented over edge devices to provide a safe and secure environment to the connected consutomer in smart cities.

## Figures and Tables

**Table 1:** Performance comparison of the proposed model with other SOTA over UCF dataset.

Method	False Alarm	AUC
AED-SC [69]	27.20	50.60
Autoencoder [21]	3.10	65.51
C3D [22]	1.90	75.41
GCN [56]	0.10	82.12
CLAWS [66]	-	83.03
DSN [8]	0.021	85.82
**Proposed**	**0.0017**	**87.55**

**Table 2:** Performance comparison of the proposed model with other SOTA over ShanghaiTech dataset.

Method	False Alarm	AUC
RNN [61]	-	68.0
FFP [9]	-	72.8
VAD-DPCN [70]	-	73.6
IPC-AD [71]	-	73.0
CAE-VAD [72]	-	73.3
VAD-SCI [60]	-	69.63
AD-MGN [17]	-	70.50
ST-AD [63]	-	73.40
GEPC-AD [62]	-	76.10
GCN [56]	-	76.44
DSFN [64]	0.74	82.14
DSN [8]	0.054	84.90
**Proposed**	**0.023**	**86.74**

**Table 3:** Performance comparison of the proposed model with other SOTA over surveillance fight dataset.

Method	False Alarm	AUC
CNN-BiLSTM [57]	-	72.0
CNN-LSTM [67]	-	74.0
CNN-ConvLSTM [68]	-	75.9
DSN [8]	0.035	93.1
**Proposed**	**0.018**	**95.8**

**Table 4:** Performance comparison of the proposed model with other SOTA over Hockey Fight dataset.

Method	False Alarm	ACC
Motion-IWLD [73]	-	96.8
HOMO-SVM [74]	-	89.3
SIFT [75]	-	96.5
3dCNN [76]	-	96.0
BOW [77]	-	95.5
CNN [78]	-	93.3
CNN [79]	-	96.4
CNN-BiLSTM [57]	-	96.0
CNN-MLSTM [67]	-	98.0
CNN-ConvLSTM [68]	-	98.5
DSN [8]	0.019	99
**Proposed**	**0.0047**	**99.8**

**Table 5:** Performance comparison of the proposed model with other SOTA over Violent Flow dataset.

Method	False Alarm	ACC
IWLD [73]	-	93.19
HOT [80]	-	82.2
HOMO-SVM [74]	-	76.8
2dCNN [76]	-	98.0
CNN-MLSTM [67]	-	98.5
DSN [8]	0.016	99.01
**Proposed**	**0.0034**	**99.81**

## Data Availability

Not applicable.

## References

[1] Skogan W.G. (2019). The future of CCTV. Criminol. Pub. Pol’y.

[2] Husman M.A., Albattah W., Abidin Z.Z., Mustafah Y.M., Kadir K., Habib S., Islam M., Khan S. (2021). Unmanned Aerial Vehicles for Crowd Monitoring and Analysis. Electronics.

[3] Chu W., Xue H., Yao C., Cai D. (2018). Sparse coding guided spatiotemporal feature learning for abnormal event detection in large videos. IEEE Trans. Multimed..

[4] Ullah W., Ullah A., Haq I.U., Muhammad K., Sajjad M., Baik S.W. (2021). CNN features with bi-directional LSTM for real-time anomaly detection in surveillance networks. Multimed. Tools Appl..

[5] Zhao B., Fei-Fei L., Xing E.P. Online detection of unusual events in videos via dynamic sparse coding. Proceedings of the Conference on Computer Vision and Pattern Recognition (CVPR 11).

[6] Rezaee K., Rezakhani S.M., Khosravi M.R., Moghimi M.K. (2021). A survey on deep learning-based real-time crowd anomaly detection for secure distributed video surveillance. Pers. Ubiquitous Comput..

[7] Ren J., Xia F., Liu Y., Lee I. (2021). Deep Video Anomaly Detection: Opportunities and Challenges.

[8] Ullah W., Hussain T., Khan Z.A., Haroon U., Baik S.W. (2022). Intelligent dual stream CNN and echo state network for anomaly detection. Knowl. Based Syst..

[9] Liu W., Luo W., Lian D., Gao S. Future frame prediction for anomaly detection–a new baseline. Proceedings of the IEEE Conference on Computer Vision and Pattern Recognition 2018.

[10] Michau G., Fink O. (2021). Unsupervised transfer learning for anomaly detection: Application to complementary operating condition transfer. Knowl. Based Syst..

[11] Nayak R., Pati U.C., Das S.K. (2021). A comprehensive review on deep learning-based methods for video anomaly detection. Image Vis. Comput..

[12] Ramachandra B., Jones M., Vatsavai R.R. (2020). A survey of single-scene video anomaly detection. IEEE Trans. Pattern Anal. Mach. Intell..

[13] Kiran B.R., Thomas D.M., Parakkal R. (2018). An overview of deep learning based methods for unsupervised and semi-supervised anomaly detection in videos. J. Imaging.

[14] Ullah W., Ullah A., Hussain T., Muhammad K., Heidari A.A., Del Ser J., Baik S.W., De Albuquerque V.H.C. (2022). Artificial Intelligence of Things-assisted two-stream neural network for anomaly detection in surveillance Big Video Data. Future Gener. Comput. Syst..

[15] Wu S., Moore B.E., Shah M. Chaotic invariants of Lagrangian particle trajectories for anomaly detection in crowded scenes. Proceedings of the 2010 IEEE Computer Society Conference on Computer Vision and Pattern Recognition.

[16] Mohammadi B., Fathy M., Sabokrou M. (2021). Image/video deep anomaly detection: A survey. arXiv Prepr..

[17] Park H., Noh J., Ham B. Learning memory-guided normality for anomaly detection. Proceedings of the IEEE/CVF Conference on Computer Vision and Pattern Recognition 2020.

[18] Albattah W., Habib S., Alsharekh M.F., Islam M., Albahli S., Dewi D.A. (2022). An Overview of the Current Challenges, Trends, and Protocols in the Field of Vehicular Communication. Electronics.

[19] Albattah W., Kaka Khel M.H., Habib S., Islam M., Khan S., Abdul Kadir K. (2020). Hajj Crowd Management Using CNN-Based Approach. Comput. Mater. Contin..

[20] Li W., Mahadevan V., Vasconcelos N. (2013). Anomaly detection and localization in crowded scenes. IEEE Trans. Pattern Anal. Mach. Intell..

[21] Hasan M., Choi J., Neumann J., Roy-Chowdhury A.K., Davis L.S. Learning temporal regularity in video sequences. Proceedings of the 2016 IEEE Conference on Computer Vision and Pattern Recognition (CVPR).

[22] Sultani W., Chen C., Shah M. (2018). Real-World Anomaly Detection in Surveillance Videos.

[23] Huang C., Li Y., Nevatia R. (2012). Multiple target tracking by learning-based hierarchical association of detection responses. IEEE Trans. Pattern Anal. Mach. Intell..

[24] Bera A., Kim S., Manocha D. Realtime anomaly detection using trajectory-level crowd behavior learning. Proceedings of the 2016 IEEE Conference on Computer Vision and Pattern Recognition Workshops (CVPRW).

[25] Singh D., Mohan C.K. (2017). Graph formulation of video activities for abnormal activity recognition. Pattern Recognit..

[26] Cheng K.-W., Chen Y.-T., Fang W.-H. (2015). Gaussian process regression-based video anomaly detection and localization with hierarchical feature representation. IEEE Trans. Image Process..

[27] Kaltsa V., Briassouli A., Kompatsiaris I., Hadjileontiadis L.J., Strintzis M.G. (2015). Swarm intelligence for detecting interesting events in crowded environments. IEEE Trans. Image Process..

[28] Zhang Y., Lu H., Zhang L., Ruan X. (2016). Combining motion and appearance cues for anomaly detection. Pattern Recognit..

[29] Colque R.V.H.M., Caetano C., de Andrade M.T.L., Schwartz W.R. (2016). Histograms of optical flow orientation and magnitude and entropy to detect anomalous events in videos. IEEE Trans. Circuits Syst. Video Technol..

[30] Alsharekh M.F., Habib S., Dewi D.A., Albattah W., Islam M., Albahli S. (2022). Improving the Efficiency of Multistep Short-Term Electricity Load Forecasting via R-CNN with ML-LSTM. Sensors.

[31] Sun L., Chen Y., Luo W., Wu H., Zhang C. Discriminative clip mining for video anomaly detection. Proceedings of the 2020 IEEE International Conference on Image Processing (ICIP).

[32] Zhu Y., Newsam S. (2019). Motion-aware feature for improved video anomaly detection. arXiv Prepr..

[33] Ullah A., Muhammad K., Haydarov K., Haq I.U., Lee M., Baik S.W. One-shot learning for surveillance anomaly recognition using siamese 3D CNN. Proceedings of the 2020 International Joint Conference on Neural Networks (IJCNN).

[34] Berroukham A., Housni K., Lahraichi M., Boulfrifi I. (2023). Deep learning-based methods for anomaly detection in video surveillance: A review. Bull. Electr. Eng. Inform..

[35] Shikalgar S., Yadav R.K., Mahalle P.N. (2023). An AI Federated System for Anomalies Detection in Videos using Convolution Neural Network Mechanism. Int. J. Intell. Syst. Appl. Eng..

[36] Taghinezhad N., Yazdi M. (2023). A new unsupervised video anomaly detection using multi-scale feature memorization and multipath temporal information prediction. IEEE Access.

[37] Kamoona A.M., Gostar A.K., Bab-Hadiashar A., Hoseinnezhad R. (2023). Multiple instance-based video anomaly detection using deep temporal encoding–decoding. Expert Syst. Appl..

[38] Chen H., Mei X., Ma Z., Wu X., Wei Y. (2023). Spatial–temporal graph attention network for video anomaly detection. Image Vis. Comput..

[39] Khan Z.A., Hussain T., Ullah F.U.M., Gupta S.K., Lee M.Y., Baik S.W. (2022). Randomly initialized CNN with densely connected stacked autoencoder for efficient fire detection. Eng. Appl. Artif. Intell..

[40] Yar H., Hussain T., Agarwal M., Khan Z.A., Gupta S.K., Baik S.W. (2022). Optimized dual fire attention network and medium-scale fire classification benchmark. IEEE Trans. Image Process..

[41] Khan K., Khan R.U., Albattah W., Nayab D., Qamar A.M., Habib S., Islam M. (2021). Crowd Counting Using End-to-End Semantic Image Segmentation. Electronics.

[42] Ullah W., Ullah A., Hussain T., Khan Z.A., Baik S.W. (2021). An efficient anomaly recognition framework using an attention residual LSTM in surveillance videos. Sensors.

[43] Khan Z.A., Hussain T., Ullah A., Rho S., Lee M., Baik S.W. (2020). Towards efficient electricity forecasting in residential and commercial buildings: A novel hybrid CNN with a LSTM-AE based framework. Sensors.

[44] Sajjad M., Khan Z.A., Ullah A., Hussain T., Ullah W., Lee M.Y., Baik S.W. (2020). A novel CNN-GRU-based hybrid approach for short-term residential load forecasting. IEEE Access.

[45] Khan Z.A., Ullah A., Ullah W., Rho S., Lee M., Baik S.W. (2020). Electrical energy prediction in residential buildings for short-term horizons using hybrid deep learning strategy. Appl. Sci..

[46] Khan Z.A., Ullah A., Haq I.U., Hamdy M., Maurod G.M., Muhammad K., Hijji M., Baik S.W. (2022). Efficient short-term electricity load forecasting for effective energy management. Sustain. Energy Technol. Assess..

[47] Muhammad K., Ullah H., Khan Z.A., Saudagar A.K.J., AlTameem A., AlKhathami M., Khan M.B., Abul Hasanat M.H., Mahmood Malik K., Hijji M. (2022). WEENet: An intelligent system for diagnosing COVID-19 and lung cancer in IoMT environments. Front. Oncol..

[48] Yar H., Imran A.S., Khan Z.A., Sajjad M., Kastrati Z. (2021). Towards smart home automation using IoT-enabled edge-computing paradigm. Sensors.

[49] Huang L., Liu G., Wang Y., Yuan H., Chen T. (2022). Fire detection in video surveillances using convolutional neural networks and wavelet transform. Eng. Appl. Artif. Intell..

[50] Yar H., Hussain T., Khan Z.A., Koundal D., Lee M.Y., Baik S.W. (2021). Vision sensor-based real-time fire detection in resource-constrained IoT environments. Comput. Intell. Neurosci..

[51] Goodfellow I., Bengio Y., Courville A. (2016). Deep Learning.

[52] Gallicchio C., Micheli A., Pedrelli L. (2017). Deep reservoir computing: A critical experimental analysis. Neurocomputing.

[53] Jaeger H. (2001). The “echo state” approach to analysing and training recurrent neural networks-with an erratum note. Bonn Ger. Ger. Natl. Res. Cent. Inf. Technol. GMD Tech. Rep..

[54] Khan Z.A., Hussain T., Baik S.W. (2022). Boosting energy harvesting via deep learning-based renewable power generation prediction. J. King Saud Univ. Sci..

[55] Khan Z.A., Hussain T., Haq I.U., Ullah F.U.M., Baik S.W. (2022). Towards efficient and effective renewable energy prediction via deep learning. Energy Rep..

[56] Zhong J.-X., Li N., Kong W., Liu S., Li T.H., Li G. Graph convolutional label noise cleaner: Train a plug-and-play action classifier for anomaly detection. Proceedings of the 2019 IEEE/CVF Conference on Computer Vision and Pattern Recognition (CVPR).

[57] Habib S., Hussain A., Islam M., Khan S., Albattah W. Towards Efficient Detection and Crowd Management for Law Enforcing Agencies. Proceedings of the IEEE 2021 1st International Conference on Artificial Intelligence and Data Analytics (CAIDA).

[58] Bermejo Nievas E., Deniz Suarez O., Bueno García G., Sukthankar R. (2011). Violence detection in video using computer vision techniques. Computer Analysis of Images and Patterns: 14th International Conference, CAIP 2011, Seville, Spain, 2–31 August 2011, Proceedings, Part II 14.

[59] Hassner T., Itcher Y., Kliper-Gross O. Violent flows: Real-time detection of violent crowd behavior. Proceedings of the 2012 IEEE Computer Society Conference on Computer Vision and Pattern Recognition Workshops.

[60] Habib S., Hussain A., Albattah W., Islam M., Khan S., Khan R.U., Khan K. (2021). Abnormal Activity Recognition from Surveillance Videos Using Convolutional Neural Network. Sensors.

[61] Luo W., Liu W., Gao S. A revisit of sparse coding based anomaly detection in stacked RNN framework. Proceedings of the 2017 IEEE International Conference on Computer Vision (ICCV).

[62] Markovitz A., Sharir G., Friedman I., Zelnik-Manor L., Avidan S. Graph embedded pose clustering for anomaly detection. Proceedings of the 2020 IEEE/CVF Conference on Computer Vision and Pattern Recognition (CVPR).

[63] Habib S., Alyahya S., Islam M., Alnajim A.M., Alabdulatif A., Alabdulatif A. (2023). Design and Implementation: An IoT-Framework-Based Automated Wastewater Irrigation System. Electronics.

[64] Yang X., Wang Z., Wu K., Xie Z., Hou J. (2021). Deep social force network for anomaly event detection. IET Image Process..

[65] Lu C., Shi J., Jia J. Abnormal event detection at 150 FPS in MATLAB. Proceedings of the 2013 IEEE International Conference on Computer Vision.

[66] Zaheer M.Z., Mahmood A., Astrid M., Lee S.-I. (2020). Claws: Clustering assisted weakly supervised learning with normalcy suppression for anomalous event detection. Computer Vision–ECCV 2020: 16th European Conference, Glasgow, UK, 23–28 August 2020, Proceedings, Part XXII 16.

[67] Farman H., Khalil A., Ahmad N., Albattah W., Khan M.A., Islam M. (2021). A Privacy Preserved, Trust Relationship (PTR) Model for Internet of Vehicles. Electronics.

[68] Ullah F.U.M., Muhammad K., Haq I.U., Khan N., Heidari A.A., Baik S.W., de Albuquerque V.H.C. (2021). AI-Assisted Edge Vision for Violence Detection in IoT-Based Industrial Surveillance Networks. IEEE Trans. Ind. Inform..

[69] Momin A.M., Ahmad I., Islam M. Weed Classification Using Two Dimensional Weed Coverage Rate (2D-WCR) for Real-Time Selective Herbicide Applications. Proceedings of the International Conference on Computing, Information and Systems Science and Engineering.

[70] Ye M., Peng X., Gan W., Wu W., Qiao Y. Anopcn: Video anomaly detection via deep predictive coding network. Proceedings of the 27th ACM International Conference on Multimedia.

[71] Tang Y., Zhao L., Zhang S., Gong C., Li G., Yang J. (2020). Integrating prediction and reconstruction for anomaly detection. Pattern Recognit. Lett..

[72] Chang Y., Tu Z., Xie W., Yuan J. (2020). Clustering driven deep autoencoder for video anomaly detection. Computer Vision–ECCV 2020: 16th European Conference, Glasgow, UK, 23-28 August 2020, Proceedings, Part XV 16.

[73] Zhang T., Jia W., He X., Yang J. (2016). Discriminative dictionary learning with motion weber local descriptor for violence detection. IEEE Trans. Circuits Syst. Video Technol..

[74] Mahmoodi J., Salajeghe A. (2019). A classification method based on optical flow for violence detection. Expert Syst. Appl..

[75] Febin I.P., Jayasree K., Joy P.T. (2020). Violence detection in videos for an intelligent surveillance system using MoBSIFT and movement filtering algorithm. Pattern Anal. Appl..

[76] Ullah F.U.M., Ullah A., Muhammad K., Haq I.U., Baik S.W. (2019). Violence detection using spatiotemporal features with 3D convolutional neural network. Sensors.

[77] Yu J., Song W., Zhou G., Hou J.-j. (2019). Violent scene detection algorithm based on kernel extreme learning machine and three-dimensional histograms of gradient orientation. Multimed. Tools Appl..

[78] Jain A., Vishwakarma D.K. Deep NeuralNet for violence detection using motion features from dynamic images. Proceedings of the 2020 Third International Conference on Smart Systems and Inventive Technology (ICSSIT).

[79] Roman D.G.C., Chávez G.C. Violence detection and localization in surveillance video. Proceedings of the 2020 33rd SIBGRAPI Conference on Graphics, Patterns and Images (SIBGRAPI).

[80] Rabiee H., Mousavi H., Nabi M., Ravanbakhsh M. (2018). Detection and localization of crowd behavior using a novel tracklet-based model. Int. J. Mach. Learn. Cybern..

